# Plant functional type composition drives summer carbon cycling in an ombrotrophic peatland

**DOI:** 10.1007/s42974-026-00357-7

**Published:** 2026-10-06

**Authors:** Agnė Bagdonaitė, Carrie L. Thomas, Yvet Telgenkamp, Bjorn J. M. Robroek

**Affiliations:** 1 Department of Ecology, Radboud Institute for Biological and Environmental Sciences, Radboud University, Heyendaalseweg 135, Nijmegen, 6525 AJ The Netherlands; 2 Department of Biology, Lund University, Kontaktvägen 10, Lund, 223 62 Sweden; 3 Department of Environmental Sciences, Radboud Institute for Biological and Environmental Sciences, Radboud University, Heyendaalseweg 135, Nijmegen, 6525 AJ The Netherlands; 4 School of Biological Sciences, Faculty of Environmental and Life Sciences, University of Southampton, Southampton, SO17 1BJ UK

**Keywords:** Carbon fluxes, Long-term plant removal experiment, Plant and microbial communities, Microhabitats, Ombrotrophic bog, Wetland

## Abstract

Peatlands are globally important carbon sinks because plant CO_2_ uptake outweighs microbial decomposition. These ecosystems are characterised by a distinct microtopography of hummocks and lawns, supporting vegetation communities composed of various plant functional types (PFTs). In this study we assess the direct effects of PFT removal and microhabitats, and the indirect effects mediated through microbial community composition on CO_2_ and CH_4_ emissions. We utilised a long-term factorial vascular plant removal experiment in a *Sphagnum*-dominated peatland in southern Sweden. We used structural equation modelling (SEM) to analyse gas flux measurements and microbial community composition, and to assess both direct and mediated effects. Plots with intact vegetation exhibited the lowest CO_2_ emissions, which increased progressively with ericoid, graminoid, and both ericoid and graminoid removal. Microhabitat type influenced microbial community structure, and only the removal of all vascular plants affected bacterial composition. However, neither CO_2_ nor CH_4_ fluxes were shown to be mediated by microbial community composition; instead, CH_4_ emissions were directly shaped by microhabitat, with higher fluxes in lawns than hummocks. These results likely reflect the timing of sampling (August), when plant-driven CO_2_ uptake dominates carbon cycling dynamics, and the limited sampling depth (~ 5 cm), which likely excluded deeper-residing microbial groups that are relevant to carbon cycling. Nevertheless, these results show that summer CO_2_ emissions are primarily driven by vegetation composition, while microhabitat, i.e. water table and oxygen, differences are more important for CH_4_ emissions.

## Introduction

Peatland ecosystems exert a disproportionate influence on the global carbon cycle. Covering just 3% of the Earth’s land surface, they store roughly a third of all soil organic carbon – more per unit area than any other wetland biome (Minasny et al. 2019; Temmink et al. 2022). The vast carbon reservoir has accumulated over millennia because persistent waterlogged, oxygen-poor conditions suppress microbial decomposition, allowing partially decomposed plant material to accumulate faster than it decays (Clymo 1984; van Breemen 1995). As a result, most intact peatlands function as long-term carbon sinks and hence maintain a net negative greenhouse gas (GHG) balance (Goodrich et al. 2017; Gallego-Sala et al. 2018). Yet this carbon sink function rests on an equilibrium that depends on a delicate balance between plant productivity, microbial decomposition, and hydrology; a balance that is increasingly threatened by environmental and climatic change (Jassey et al. 2018; Barel and Robroek 2023).

Northern ombrotrophic peatlands are particularly effective carbon sinks (Yu et al. 2012; Temmink et al. 2022). Solely fed by rainwater, their acidic, nutrient-poor, soils support a unique and specialised plant community and associated microbiome, that together promote carbon accumulation. The peatland plant community is structured around three main plant functional types (PFTs): *Sphagnum* mosses, ericoid shrubs, and graminoids (van Breemen 1995). Moreover, the dominance of *Sphagnum* mosses is central to the carbon sink function of peatlands: their recalcitrant tissues and the release of phenolic acids suppress organic matter decomposition trough it effects on microbial and enzymatic activity (McGivern et al. 2024; Birch and Bell 2026),. Vascular plants, though less abundant, also play important roles in carbon cycling. Through photosynthesis, they contribute directly to CO₂ uptake (Kuiper et al. 2014), and at low cover they can facilitate *Sphagnum* growth by reducing evapotranspiration (Heijmans et al. 2001), stabilising the microclimate (Buttler et al. 1998), and by providing structural support (Telgenkamp et al. 2025).

Environmental and climatic change is increasingly altering the composition of peatland plant communities (Robroek et al. 2017; Lamentowicz et al. 2019). Warming and changing precipitation regimes – notably increased summer droughts – promote the transition from *Sphagnum*-dominated vegetation toward greater abundance of ericoid shrubs and graminoids (Dieleman et al. 2015; Antala et al. 2022; Gong et al. 2024). Such shifts in vegetation cascade through the ecosystem. As plant assemblages change, so do the microbial communities they interact with (Robroek et al. 2021). Yet, the combined effects of these concerted (plant-microbe) shifts on carbon dynamics remain poorly understood. At its core, this is however a fundamental ecological question as ericoid shrubs and graminoids differ markedly in their physiology, litter quality, and microbial associations (Dorrepaal et al. 2005; Robroek et al. 2015). Moreover, how these differences scale up to govern peatland carbon cycling is not well resolved. Resolving this question is relevant as climate warming is projected to favour vascular plants over *Sphagnum* mosses in many peatlands, making the consequences of shifting PFT dominance for carbon dynamics a pressing applied concern as well.

An important pathway through which different PFTs influence peatland carbon cycling is by shaping the structure and activity of the microbial community (Robroek et al. 2015). *Sphagnum* mosses host methanotrophic bacteria that act as a filter for CH₄ emissions (Kip et al. 2010; Stępniewska et al. 2018) and photoautotrophic microbes that contribute to CO_2_ uptake (Hamard et al. 2025; Kuuri-Riutta et al. 2025). Ericoid shrubs associate with ericoid mycorrhizal fungi, which promote carbon storage through several mechanisms: they compete with saprotrophic fungi (Bödeker et al. 2016), suppress syntrophic bacteria and methanogens (Chroňáková et al. 2019), and produce melanin-rich recalcitrant biomass and necromass that stabilises soil carbon (Averill and Hawkes 2016; Fernandez et al. 2019; Könönen et al. 2026). Graminoids, in contrast, tend to stimulate microbial activity by exuding labile carbon compounds (Mastný et al. 2018), producing easily degradable litter, and increasing radial oxygen loss; all of which elevate decomposition (Mao et al. 2018; Bhullar et al. 2013). Their aerenchymous tissues further facilitate diffusive CH₄ transport from deeper peat layers, effectively bypassing the methanotrophic CH_4_ oxidation filter and resulting in elevated emissions (Dieleman et al. 2017; Gong et al. 2021; Zhang et al. 2022). In peatlands, these plant-driven effects on microbial communities are modulated by microhabitat structure, which creates distinct gradients in water and oxygen availability. In lawns, higher water tables and anoxic conditions favour methanogenic archaea and anaerobic bacteria (Chroňáková et al. 2019), increasing CH_4_ production while reducing aerobic decomposition. In the drier, more oxygenated hummocks, aerobic microorganisms such as fungi, methanotrophs, and other decomposers are more abundant (Robroek et al. 2015; Iseas et al. 2025) and can promote CO_2_ emissions.

Despite growing recognition that vegetation shifts can reshape peatland carbon cycling, empirical evidence linking plant community change to microbial responses and greenhouse gas fluxes remains limited. Where such links have been studied, investigations have typically been short-term or have compared spatially distant communities, making it difficult to disentangle the direct effects of climate from those mediated by vegetation change (Guo and Wang 2026). Overcoming this limitation requires controlled, long-term experiments where plant community composition is manipulated independently of climate. Here, we use a long-term plant removal experiment to address the role of plant functional type (PFT) composition in peatland carbon dynamics. Specifically, we determine the direct and indirect effects of PFTs, microhabitat, and microbial community composition on CO₂ and CH₄ fluxes. We hypothesised that (i) removal of vascular PFTs would reduce CO₂ uptake, with the strongest reduction following combined ericoid and graminoid removal; (ii) microhabitat would be the primary control on CH₄ emissions, given its direct link to water table depth; and (iii) PFT and microhabitat effects on carbon fluxes are partly mediated by shifts in microbial community composition.

## Materials and methods

### Study site

Store Mosse National Park (57°17’54 N, 14°00’19E; 166 m a.s.l.), covering approximately 7,740 ha, represents the largest intact peatland complex in south-central Sweden (Kylander et al. 2023). The landscape originated after the retreat of the Weichselian ice sheet (~ 14 ka BP), when sub-basins of varying depth, interspersed with aeolian sand dunes, underwent terrestrialisation, giving rise to a mosaic of lakes, fens, and bogs. The undisturbed bog surfaces feature a well-developed *Sphagnum* carpet with pronounced hollow-lawn-hummock microtopography, dominated by *S. cuspidatum*, *S. balticum*, *S. medium*, *S. rubellum*, and *S. fuscum*. The vascular flora includes the graminoids *Eriophorum vaginatum*, *Trichophorum cespitosum*, and *Rhynchospora alba*, alongside the ericaceous dwarf shrubs *Calluna vulgaris*, *Erica tetralix*, and *Vaccinium oxycoccos*. Other characteristic species are *Drosera rotundifolia*, *D. anglica*, *Rubus chamaemorus*, and *Cladonia* spp. (predominantly *C. portentosa* and *C. arbuscula*).

### Experimental setup

**Fig. 1:**
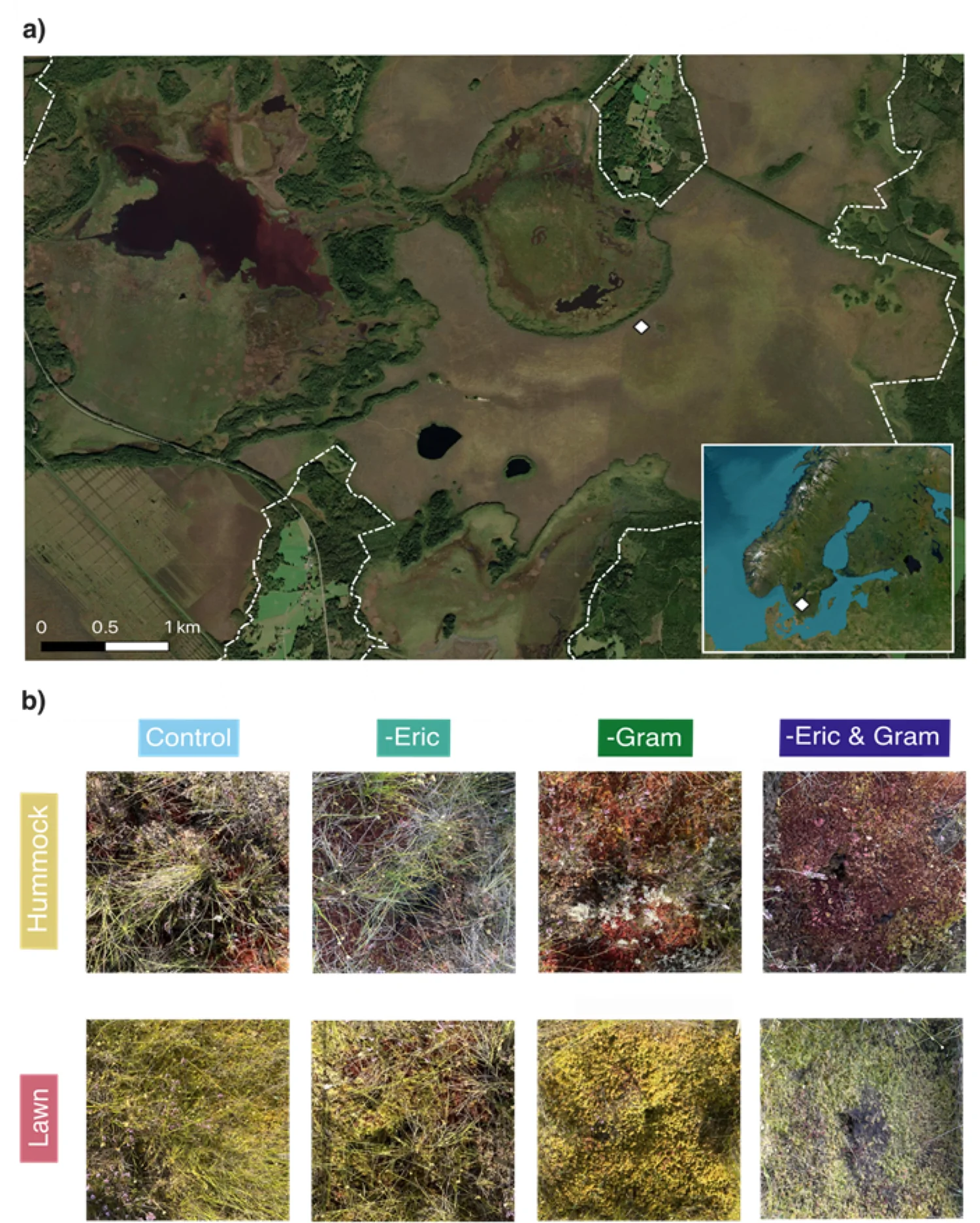
**a** Location of experimental site (white diamond) within Store Mosse National Park and Sweden. Dashed white line indicates boundary of Store Mosse National Park. **b** Photos of a representative plot for each treatment in both microhabitatats (Hummocks and Lawns): Control, ericoid removal (– Eric), graminoid removal (– Gram) or ericoid and graminoid removal (– Eric & Gram) treatment. Colour scheme corrensponds to colours in Figs. 2, 4 and 5

We utilised a long-term plant functional type removal experiment situated in a *Sphagnum*-dominated peatland within the national park (Fig. 1a) to investigate how vascular plant composition influences microbial community assembly and carbon dynamics. The experiment was established in the summer of 2011 and consists of 40 plots (50 × 50 cm), divided in 20 lawn and 20 hummocks plots, arranged across five blocks. Lawns were persistently near-saturated (water table within a few cm of the surface) and hummock water table depth remained at 20–30 cm below the *Sphagnum* surface. All plots were within one hectare of the peatland. Within each block, the vascular plant community was manipulated in both microhabitats using the following treatments: removal of ericoids (− Eric), removal of graminoids (− Gram), removal of both ericoids and graminoids (− Eric & Gram), and a non-manipulated control (Fig. 1b). At the onset of the experiment, target vascular plants (see Table 1 for a microhabitat specific description of the original plant community composition) were clipped flush with the *Sphagnum* surface, and plots have since been maintained at least three times per year by uprooting any re-establishing seedlings. Forb cover within plots was negligible (< 1% cover on average) in all plots. Prior to treatment establishment, plant community composition was similar within and across blocks (see Robroek et al. 2015 and Telgenkamp et al. 2025 for a full vegetation survey of the experimental site).

**Table 1:** Composition of the plant community in the experimental area in the Store Mosse National Park (after Robroek et al. 2015)

Dominant vegetation	Lawn	Hummock
Mosses	*Sphagnum cuspidatum*	*Sphagnum medium* *Sphagnum rubellum*
Ericoids	*Erica tetralix* *Vaccinium oxycoccus* *Andromeda polifolia*	*Erica tetralix* *Vaccinium oxycoccus* *Andromeda polifolia* *Empetrum nigrum* *Calluna vulgaris*
Graminoids	*Eriophorum angustifolium* *Rhynchospora alba*	*Eriophorum vaginatum*

### Carbon dioxide and methane—measurements and data processing

Carbon dioxide (CO₂) and methane (CH₄) fluxes were measured across all 40 plots in August 2024 using a static chamber approach coupled to an ultra-portable greenhouse gas analyser (ABB – Los Gatos Research, San Jose, CA, USA). Three successive measurement campaigns were conducted within a single week to capture short-term temporal variability in carbon dynamics, with all three campaigns conducted at approximately the same time of day (late morning, 09:00–12:00) to minimise diurnal variation. A transparent acrylic chamber (30 × 30 cm, 40 cm height) was placed onto a permanently installed 30 × 30 cm collar located at the centre of each plot and connected in a closed loop. For every plot, two types of gas measurement with a 1hz frequency were conducted over a 180 s interval: ‘light’ measurements and ‘dark’ measurements. ‘Light’ measurements with the transparent chamber represent net ecosystem exchange (NEE), while ‘dark’ measurements – where we used an opaque cover over the chamber – represents ecosystem respiration (Reco). Chamber air temperature was logged with a HOBO Onset recorder (Onset Computer Corporation, Bourne, MA, USA) to correct for temperature increases caused by solar heating. Photosynthetically Active Radiation measurements were recorded from the on-site weather station.

$$F = ~\frac{{P~ \times \phi \times slope}}{{R~ \times T}}$$

Rates of change in CO_2_ or CH_4_ concentration (ppm s⁻¹ m⁻²) were obtained using a robust linear regression with the *gasfluxes* R package (Fuss 2024). Only the most linear segment of each 180 s measurement was used to define the slope, resulting in 90–180 s intervals. Fluxes (F, μmol m⁻² s⁻¹) were then calculated using the ideal gas law:

Where P = atmospheric pressure (kPa), ϕ = the humidity correction factor (1 − 10⁶ H₂O), the slope = the rate of concentration change (ppm s⁻¹), R = the universal gas constant (8.314 J mol⁻¹ K⁻¹), and T = chamber air temperature (K). Fluxes were additionally corrected for chamber headspace volume and normalised to the collar basal area. One CO_2_ and CH_4_ flux measurement was taken for each plot per day, resulting in three flux measurements per plot in total. Net ecosystem exchange (NEE) and CH_4_ fluxes were calculated by averaging the light measurements. We used the ecological sign convention for the flux data; hence, positive flux values indicate uptake, while negative flux values indicate loss of CO_2_ and CH_4_ to the atmosphere.

### Microbial sampling and molecular methods

Peat samples were collected at a *c.* 5 cm depth below the *Sphagnum* surface of each plot, immediately outside the permanent flux collar (within *c.* 10 cm), to avoid disturbance for the CO_2_ and CH_4_ flux measurement, but within the 50 × 50 cm PFT treatment plots. Samples were kept cool at 6 °C during transport and then stored at −20 °C until DNA extraction. The DNA was extracted from 0.4 g sample using the Qiagen DNeasy PowerSoil Pro Kit according to the manufacturer protocol. Bacterial and fungal community compositions were characterised through amplicon sequencing targeting the 16 S rRNA and ITS2 regions, respectively. For the prokaryotes, the V4 region of the 16 S rRNA gene was amplified using primers 515 F (GTGCCAGCMGCCGCGGTAA) and 806R (GGACTACHVGGGTWTCTAAT). For fungi, the ITS2 region was amplified using primers ITS3F (GCATCGATGAAGAACGCAGC) and ITS4R (TCCTCCGCTTATTGATATGC). All sequencing was performed by Novogene Co., Ltd. (Beijing, China) using the Illumina NovaSeq 6000 platform with a paired-end 2 × 250 bp configuration.

Amplicon sequence variants (ASVs) were identified using the DADA2 algorithm (Callahan et al. 2016), including quality filtering, denoising and removal of chimeric sequences. Taxa were assigned within DADA2 using the SILVA database (release 138) (Glöckner 2019) for 16 S rRNA gene sequences and the UNITE database (version 19.02.2025, (Abarenkov et al. 2024) for ITS2 sequences. To improve taxonomic resolution at the order level, the 50 most abundant unassigned ASVs (out of 888) were queried using the BLAST algorithm (Altschul et al. 1990) against the core nucleotide (core_nt) database at the National Center for Biotechnology Information (NCBI). Taxonomic assignments were made based on the following criteria: (i) 100% query coverage, (ii) sequence identity > 90%, and (iii) top hits originating from cultured isolates. This approach enabled the assignment of approximately 80% of previously unclassified ASVs at the order level.

Reads assigned as mitochondrial, chloroplast or other non-bacterial sequences for bacterial data, and non-fungal sequences for fungal data, were filtered out using the *microeco* package (Liu et al. 2026) in R. Archaeal ASVS were retained during denoising and taxonomic assignment but excluded from downstream community ordinations and SEM because archaeal reads were rare and highly sparse across samples (5480 sequences in total; ~0.16% of prokaryotic reads, median per-sample 0.0105%), limiting robust inference on community structure, despite the importance of archaea in methane cycling.

### Data analysis

To obtain total carbon emissions, CH₄ fluxes were converted into CO₂ equivalents using a global warming potential based on the 100-year horizon (GWP-100; IPCC 2021) and added to the measured CO₂ emissions. For each plot, the three repeated flux measurements were averaged prior to analysis, yielding a single NEE and CH₄ value per plot (*n* = 40; 5 blocks × 4 PFT treatments × 2 microhabitats, i.e. *n* = 5 per PFT treatment × microhabitat combination). Two-way ANOVAs (PFT treatment × microhabitat, including their interaction), with block as an additional blocking factor, were used to test effects on NEE, CH₄, and total C flux, followed by Tukey’s post hoc tests when main effects were significant.

Fungal and bacterial community compositions were transformed into Aitchison distance matrices using the *compositions* R package (van den Boogaart and Tolosana-Delgado 2008) to account for the compositional nature of microbiome data (Gloor et al. 2017). Principal Coordinates Analysis (PCoA) was then conducted with the *microeco* R package to reduce the dimensionality of the data. Scores from the first principal coordinate axis (PCo1) were extracted to represent microbial community structure in subsequent analyses. PCo1 was used as a univariate summary of community composition because it was the only axis that, for both bacteria and fungi, loaded primarily on microhabitat. This approach made it a tractable and ecologically interpretable single predictor for inclusion in the SEM.

Structural equation modelling (SEM) was used to assess the direct and indirect (mediation) effects of microhabitat type, plant functional type treatment, and microbial composition on total carbon emissions. Separate SEMs were developed for fungal and bacterial communities, using the *lavaan* package in R (Rosseel 2012). To include categorical predictors in the SEM, microhabitat and each treatment level were converted into binary variables (i.e., distinct variables for microhabitat, −Eric, −Gram, and −Eric & Gram). The mediation effects of microbial community composition were calculated as the product of regression coefficients for paths *treatment/microhabitat → PCo1* and *PCo1 → CH*_*4*_ */CO*_*2*_ *emissions*. No global goodness-of-fit measure could be obtained as the SEMs were just-identified (df = 0). Hence, all theoretically motivated paths were included, leaving no residual degrees of freedom to assess model fit. Path coefficients and their significance remain interpretable, and model specification was guided a priori by known plant–microbe–carbon relationships. We found block not to affect the models and did therefore not include block as a random effect in the SEM. All statistical analyses were performed in the R environment (RStudio v.4.6.0).

## Results

### Carbon dioxide and methane fluxes

**Fig. 2:**
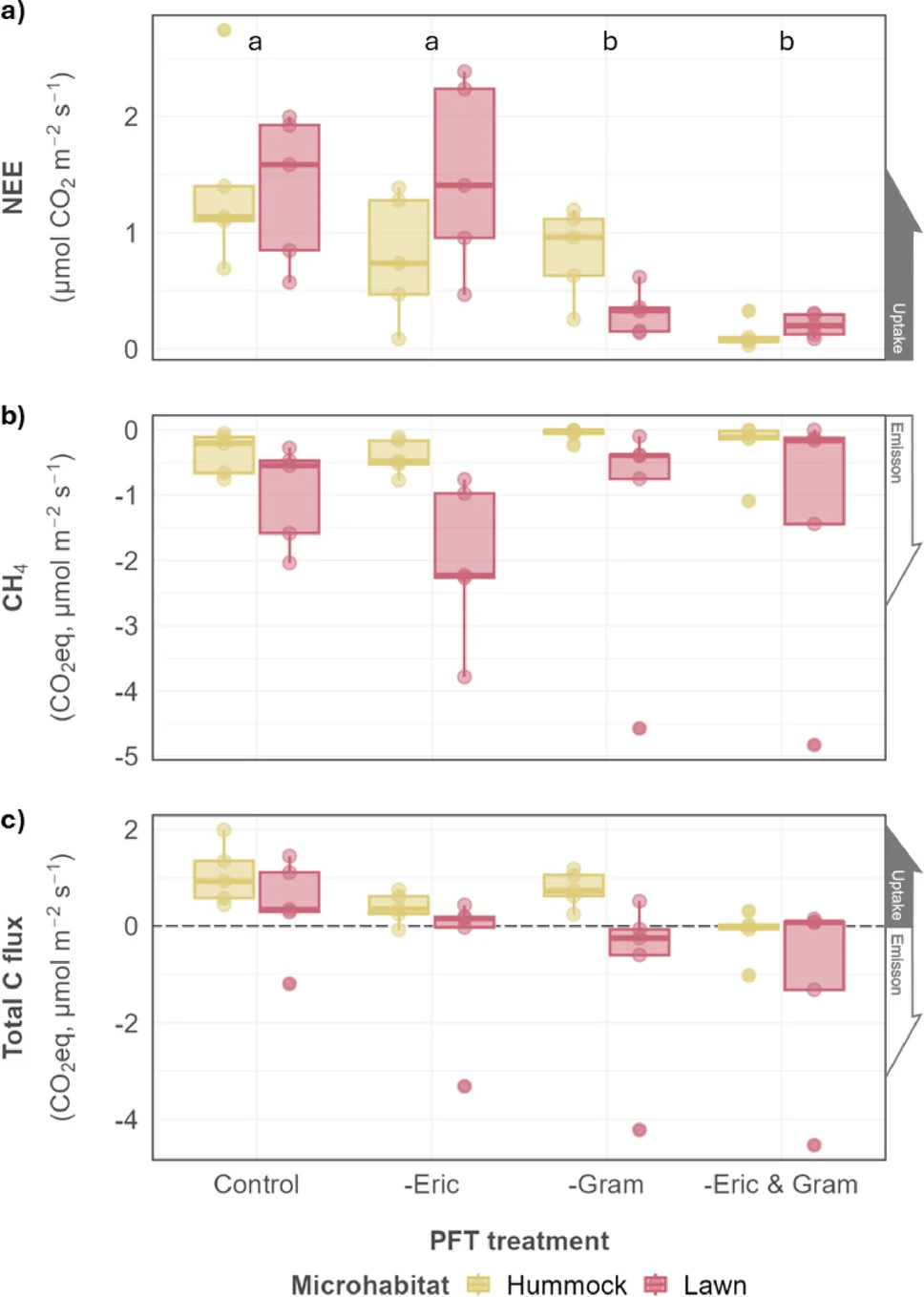
Net Ecosystem Exchange (NEE) (**a**), CH_4_ (**b**), and total carbon fluxes (CO_2_ + CH_4_) in CO_2_ equivalents (**c**) across PFT treatments and microhabitats (all in µmol m^−2^s^− 1^). Different letters denote significant differences between PFT treatments at α = 0.05. A dashed horizontal line at zero is added to each panel, with axis labels indicating the direction of net uptake versus net emission to the atmosphere

Net ecosystem exchange (NEE) was positive across all plots (range: 0.03 to 2.74 µmol m^− 2^ s^− 1^; mean = 0.82 µmol m^− 2^ s^− 1^), indicating net CO_2_ uptake, but decreased through –Eric, –Gram, to –Eric & Gram treatments (Fig. 2a). Microhabitat did not affect NEE overall (F_1,31_ = 0.13, *P* = 0.72). However, a marginal interaction between PFT treatment and microhabitat (F_3,31_ = 2.24, *P* = 0.10) suggests that the effect of PFT removal depended on microhabitat, consistent with the dominance of graminoids in lawns and ericoid shrubs in hummocks. Accordingly, removal effects on NEE were greatest where the targeted functional type naturally contributed most to plant community composition. CH₄ emissions differed between microhabitats (F_1,31_ = 9.82, *P* = 0.004), with larger net CH₄ loss (i.e. greater emission, following our sign convention where negative values denote efflux) in lawns (mean = −1.38 ± 1.49 µmol m^− 2^ s^− 1^) than hummocks (mean = −0.28 ± 0.32 µmol m^− 2^ s^− 1^; Fig. 2b), whereas PFT removal treatments had no effect on CH_4_ fluxes (F_3,31_ = 9.82, *P* = 0.66). Total carbon fluxes expressed as CO_2_ equivalents (CO_2−eq_) were not significantly different from zero (range: −4.53 to 1.99 µmol m⁻² s⁻¹; mean = −0.01 µmol m⁻² s⁻¹; one-sample t-test, *P* = 0.96; Fig. 2c) and followed a pattern broadly similar to NEE, although the effects of PFT removal were not significant here. Microhabitat significantly affected total carbon fluxes (F_1,31_ = 7.69, *P* = 0.01), with lawns showing higher emissions than hummocks, while no interaction between PFT treatment and microhabitat was detected (F_3,31_ = 0.35, *P* = 0.79).

### Microbial community composition

**Fig. 3:**
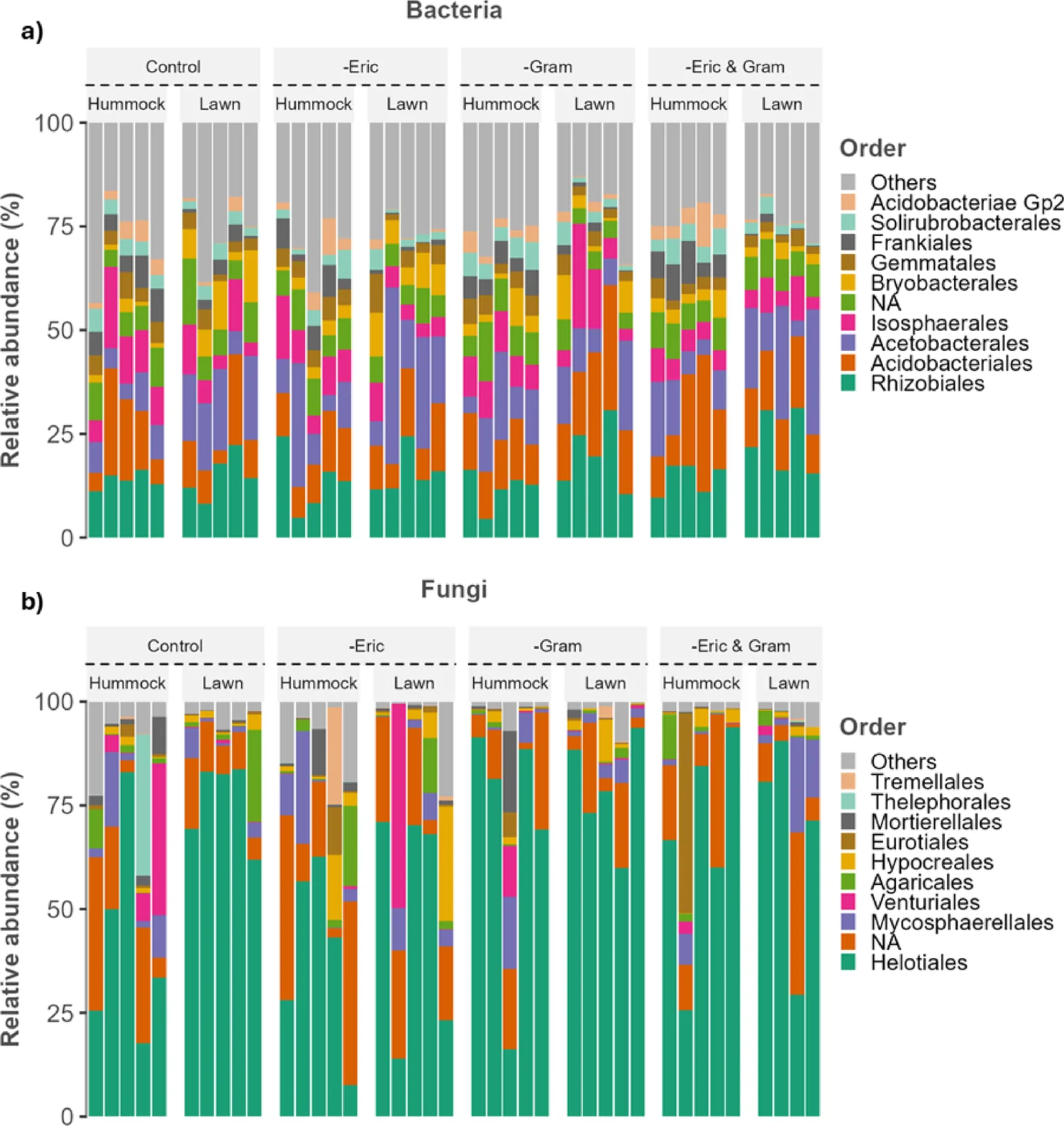
The relative abundances of **a** bacterial and **b** fungal taxa at order level across PFT treatments and microhabitats

The bacterial community dataset comprised 3,412,315 sequences, with per-sample read counts ranging between 39,399 and 126,384 (mean = 85,307 ± 18,776), and ASVs richness ranging from 861 to 2,141 per sample (mean = 1,314 ± 263). The fungal dataset contained 1,336,025 sequences, with per-sample read counts ranging from 4790 to 80,829 (mean = 34,257 ± 16,488) and ASV richness ranging from 25 to 100 per sample (mean = 62 ± 17). The lower fungal read depth and ASV richness, relative to bacteria, likely reflects the inherently lower fungal diversity that is typical for ombrotrophic bogs (Fig. 3). Within the bacterial community, *Rhizobiales*, *Acidobacteriales*, and *Acetobacteriales* were the dominant orders, consistent with the acidic, nutrient-poor conditions that characterise ombrotrophic bogs. Moreover, bacterial community composition was broadly similar across PFT removal treatments, with within-PFT treatment variability masking any apparent treatment signal (Fig. 3). The fungal community was overwhelmingly dominated by *Helotiales* across all treatments and microhabitats, with no distinct treatment-related shift in relative abundance (Fig. 3).

**Fig. 4:**
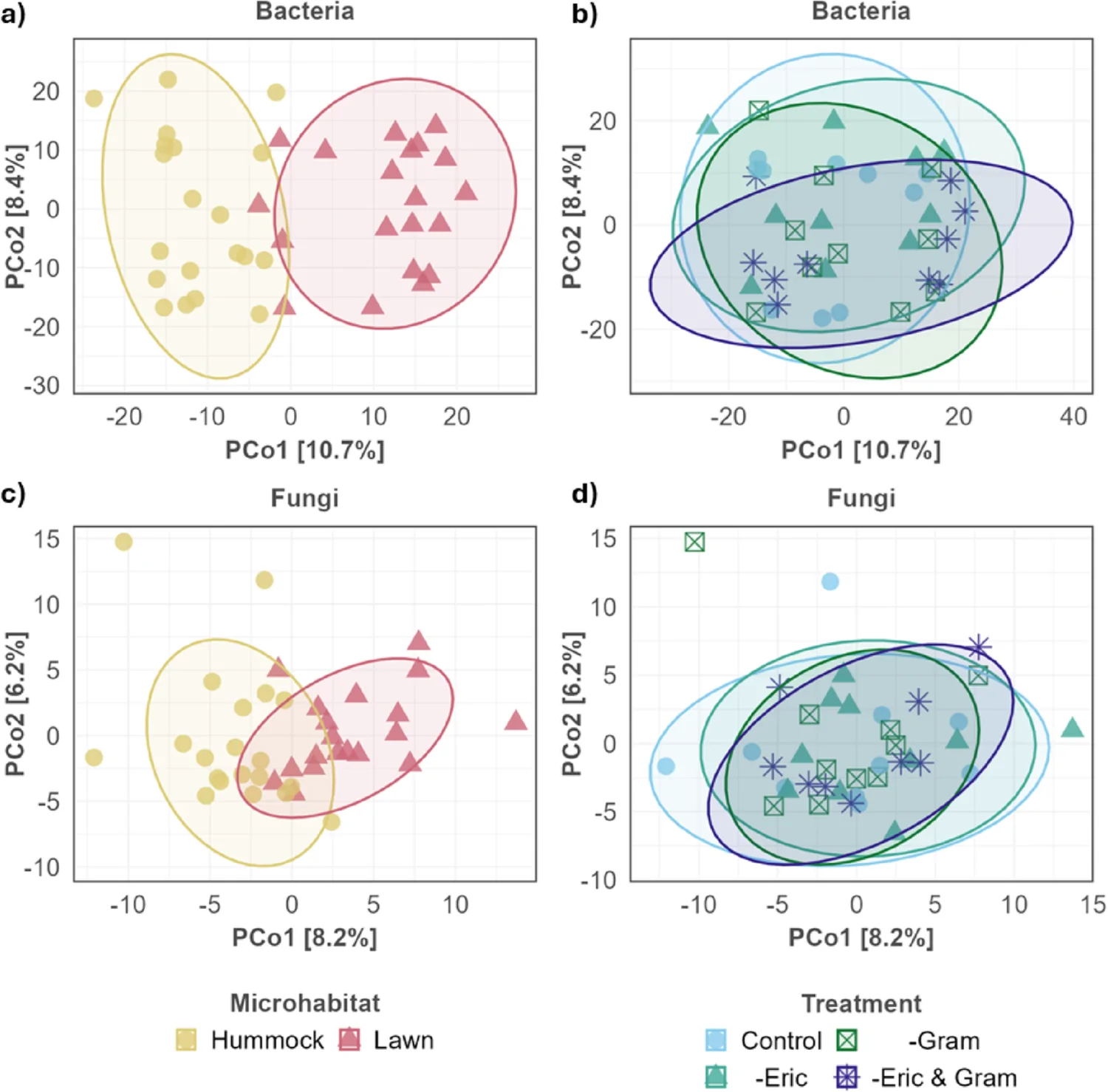
Principal Coordination Analysis plots of the bacterial (**a**, **b**) and fungal (**c**, **d**) community variation between microhabitats and PFT treatments

Community composition of bacteria and fungi was examined separately using Principal Coordinates Analysis (PCoA; Fig. 4). For bacteria, PCo1 and PCo2 explained 10.7% and 8.4% of total variation, respectively, with microhabitat emerging as the primary driver of community separation along PCo1. For fungi, PCo1 and PCo2 accounted for 8.2% and 6.2% of variation, respectively, with a similar though less pronounced microhabitat signal.

### Structural equation modelling

**Fig. 5:**
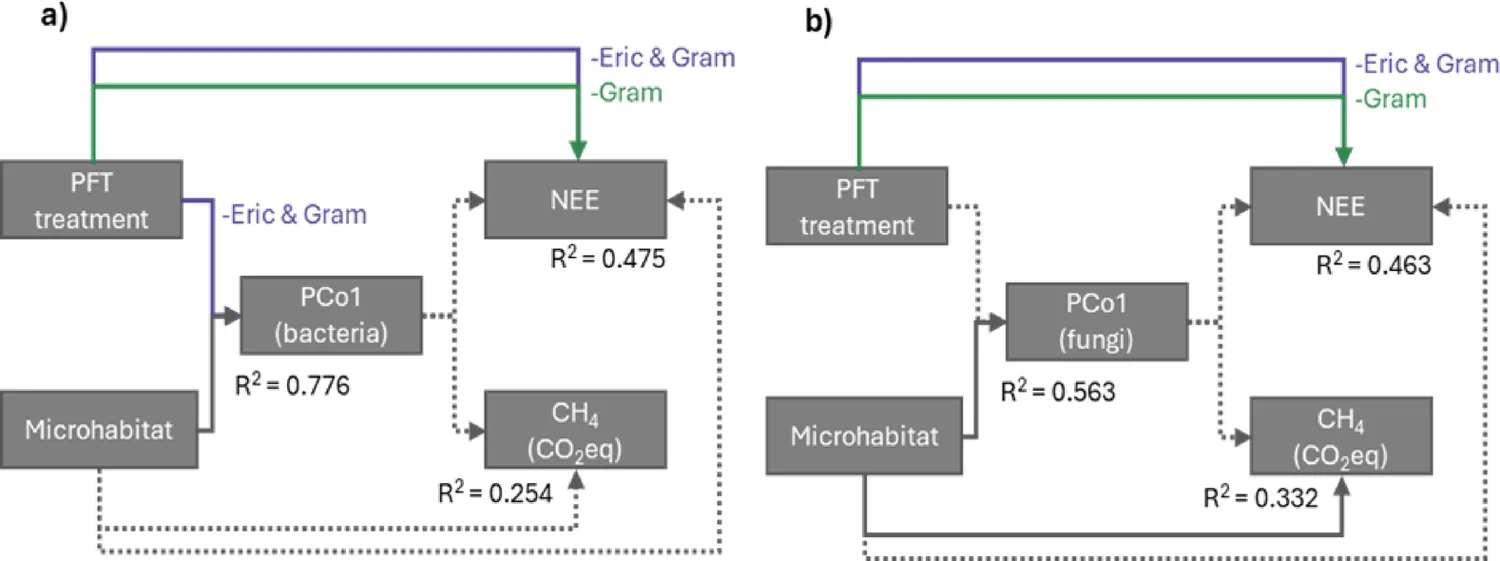
Structural equation models (SEMs) illustrating the direct and indirect effects of plant functional type (PFT) removal treatments and microhabitat on net ecosystem exchange (NEE; µmol m^− 2^ s^− 1^) and CH_4_ emissions (CO_2−eq_ µmol m^− 2^ s^− 1^) via microbial community composition (PCo1). **a** shows the model using bacterial community data; **b** uses fungal community data. Solid arrows represent statistically significant paths (*p* < 0.05), while dashed arrows indicate non-significant relationships. For clarity, only PFT treatment paths with significant effects are shown

Direct and indirect effects of PFT removal, microhabitat, and microbial community composition on carbon fluxes were evaluated using separate structural equation models for the bacterial and fungal communities (Fig. 5; Table 2). Microhabitat structured both the bacterial and fungal communities (*p* < 0.001), though treatment and microhabitat together explained a substantially greater proportion of variation in bacterial community composition (R² = 0.776) than in fungal composition (R² = 0.563), indicating a stronger plant–bacteria coupling in our plots. Among PFT removal treatments, only −Eric & Gram had a detectable effect on microbial community composition, and this was restricted to the bacterial model (*P* = 0.03). In both models, the removal of graminoids (− Gram) and the removal of both functional types (− Eric & Gram) had detectable direct effects on NEE (*p* < 0.001), while microhabitat had no direct effect on NEE in either model. Microbial community composition (PCo1) did not influence NEE, and no indirect effects on NEE mediated through microbial composition were detected for any treatment.

**Table 2:** Summary of the structural equation models (SEM) assessing the direct, indirect and total effects of microhabitat and PFT removal treatments on net ecosystem exchange (NEE; µmol m^− 2^ s^− 1^) and CH_4_ emissions (CO_2 − eq_ µmol m^− 2^ s^− 1^)

Parameter	Bacteria				Fungi			
	Estimate	Std. Error	z-value	p-value	Estimate	Std. Error	z-value	p-value
Effects on PCo1								
Microhabitat	22.539	1.948	11.571	**<0.001**	7.299	1.064	6.859	**<0.001**
Eric	2.783	2.755	1.01	0.312	2.219	1.484	1.495	0.135
Gram	3.879	2.755	1.408	0.159	−0.106	1.484	−0.071	0.943
Eric & gram	5.967	2.755	2.166	**0.03**	1.547	1.526	1.014	0.311
Direct effects on NEE								
Microhabitat	0.187	0.339	0.553	0.58	−0.201	0.248	−0.809	0.419
Eric	0.289	0.233	1.243	0.214	0.216	0.239	0.901	0.368
Gram	0.866	0.235	3.683	**<0.001**	0.826	0.233	3.547	**<0.001**
Eric & gram	1.303	0.243	5.367	**<0.001**	1.208	0.243	4.983	**<0.001**
PCo1	−0.011	0.013	−0.833	0.405	0.019	0.025	0.767	0.443
Indirect effects (via PCo1) on NEE								
Microhabitat × PCo1	−0.248	0.298	−0.831	0.406	0.141	0.185	0.763	0.446
Eric × PCo1	−0.031	0.048	−0.643	0.52	0.043	0.063	0.683	0.495
Gram × PCo1	−0.043	0.059	−0.717	0.473	−0.002	0.029	−0.071	0.943
Eric & gram × PCo1	−0.066	0.084	−0.778	0.437	0.03	0.049	0.612	0.541
Total effects (direct + indirect) on NEE								
Microhabitat	−0.06	0.164	−0.368	0.713	−0.06	0.168	−0.356	0.722
Eric	0.258	0.232	1.116	0.264	0.258	0.235	1.102	0.27
Gram	0.824	0.232	3.557	**<0.001**	0.824	0.235	3.512	**<0.001**
Eric & gram	1.237	0.232	5.342	**<0.001**	1.238	0.241	5.134	**<0.001**
Direct effects on CH₄ (CO₂eq)								
Microhabitat	1.128	0.675	1.67	0.095	1.792	0.465	3.851	**<0.001**
Eric	0.539	0.464	1.162	0.245	0.723	0.449	1.61	0.107
Gram	−0.013	0.469	−0.028	0.978	−0.026	0.437	−0.059	0.953
Eric & gram	0.128	0.484	0.263	0.792	0.392	0.455	0.862	0.389
PCo1	−0.001	0.026	−0.035	0.972	−0.084	0.047	−1.788	0.074
Indirect effects (via PCo1) on CH₄ (CO₂eq)								
Microhabitat × PCo1	−0.021	0.593	−0.035	0.972	−0.615	0.356	−1.73	0.084
Eric × PCo1	−0.003	0.073	−0.035	0.972	−0.187	0.163	−1.147	0.251
Gram × PCo1	−0.004	0.102	−0.035	0.972	0.009	0.125	0.071	0.943
Eric & gram × PCo1	−0.006	0.157	−0.035	0.972	−0.13	0.148	−0.882	0.378
Total effects (direct + indirect) on CH₄ (CO₂eq)								
Microhabitat	1.107	0.324	3.418	**0.001**	1.177	0.326	3.612	**<0.001**
Eric	0.536	0.458	1.171	0.242	0.536	0.454	1.18	0.238
Gram	−0.017	0.458	−0.037	0.971	−0.017	0.454	−0.037	0.97
Eric & gram	0.122	0.458	0.266	0.79	0.262	0.467	0.56	0.575

Results are shown for both bacterial and fungal datasets, with microbial community composition represented by PCo1. Numbers in bold indicate significant (P ≤ 0.05) effects.

For CH_4_ fluxes (expressed as CO_2−eq_), microhabitat exerted a significant total effect in both models (bacteria: *p* = 0.001; fungi: *P* < 0.001), yet no evidence of mediation through microbial community composition was found in either. Notably, microhabitat showed a significant direct effect on CH₄ emissions in the fungal model (*P* < 0.001) but not in the bacterial model, suggesting that the bacterial community may have partially mediated the microhabitat signal. PFT removal treatments had no effect on CH_4_ fluxes in either model.

## Discussion

The net neutral carbon balance across treatments that we observe, combined with the progressive increase in CO₂ loss following PFT removal, confirms an important role of vascular plant photosynthesis in driving summer carbon uptake at this ombrotrophic bog. This is consistent with earlier work showing that vascular plant functional types define the magnitude of peatland CO₂ exchange (Kuiper et al. 2014; Gavazov et al. 2018; Gong et al. 2024), and with observations that PFT diversity sustains carbon-related multifunctionality in peatlands (Robroek et al. 2017). The marginal interaction between PFT treatment and microhabitat (*p* = 0.094) is consistent with the established niche preferences of ericoids in hummocks and graminoids in lawns (Robroek et al. 2015; Telgenkamp et al. 2025), suggesting that removal effects on NEE are strongest where the removed functional type was dominant.

Despite the well-documented role of graminoids in promoting CH_4_ production and transport via aerenchymatous tissue and root exudates (Bhullar et al. 2013; Zhang et al. 2022; Määttä and Malhotra 2024), graminoid removal had no detectable effect on CH₄ fluxes. This is consistent with evidence that at low water tables, plant functional type composition loses its influence on CH₄ fluxes, as the depth of the anaerobic zone becomes the primary control on methanogenesis (Riutta et al. 2020; Whitaker et al. 2021). Instead, microhabitat was the dominant driver of CH₄ emissions, with lawns emitting significantly more CH_4_ than hummocks, reflecting the persistently higher water tables and more strongly anaerobic conditions in the former (Chroňáková et al. 2019; Robroek et al. 2015). Although vegetation and microtopography may contribute to local variation in CH₄ emissions, we consider differences in water-table depth between the microhabitat conditions the most parsimonious explanation for the observed CH₄ pattern. This interpretation alignes with syntheses that identify water-table position as the dominant control on peatland methane fluxes (Le Mer and Roger 2001; Turetsky et al. 2014), and with a more recent large-scale analysis thar demonstrates that water-table depth overrides many other environmental controls on peatland greenhouse-gas exchange (Evans et al. 2021).

Microhabitat had no direct effect on CO_2_ fluxes, following our expectations and previous work (Krohn et al. 2017), as microhabitat itself does not act as a direct source or sink of carbon. Additionally, microhabitat affected the bacterial and fungal communities as expected, due to the differences of lawn and hummock habitats in terms of water table and oxygen conditions (Zhang et al. 2024). Among the PFT removal treatments, only the ‘–Eric & Gram’ condition significantly altered the bacterial community composition. This result is plausible given that the removal of both ericoids and graminoids represents the most severe deviation from the natural (control) community structure. However, the absence of a PFT removal effect on the fungal community contrasts with previous findings, as PFTs have been shown to affect microbial community indirectly, through root exudates, as well as directly through plant-microbe associations, such as ericoid shrubs with ericoid mycorrhizal fungi (Robroek et al. 2015; Lamit et al. 2021). This difference may partly be due to methodological differences between studies. Lamit et al. (2021) and Robroek et al. (2015) used peat mesocosms, where fungi cannot disperse in from surrounding areas, which may have made community shifts easier to detect. Additionally, both studies ran for only 2–3 years, meaning the fungal community changes they observed may partly reflect the disturbance of removing plants rather than the long-term absence of the PFT. In our field experiment, 13 years is likely sufficient for any such disturbance effects to have faded. This points to a broader, largely open question of how resilient peatland microbial communities are to sustained plant community change, and over what timescale apparent ‘recovery’ (i.e. convergence of treatment and control communities, as our results seem to point to for fungi) reflects true functional resilience rather than a shift to an alternative stable state. We did not characterise microbial communities at intermediate time points over the 13-year course of the experiment (but see Robroek et al. 2015), and addressing this question directly would require a temporal series of microbial sampling across the treatment history, which we identify as a priority for future work at this site.

Neither bacterial nor fungal community composition mediated the effects of PFT removal or microhabitat on CO_2_ or CH_4_ fluxes. For CO_2_, this is not unexpected given that measurements in August capture peak growing-season conditions when plant photosynthetic uptake overrides any microbial driven decomposition signal (Korrensalo et al. 2020). The apparent absence of microbial mediation of CH₄ fluxes seems less straightforwardly, given that summer is typically the period of peak methane production and emission (Goodrich et al. 2011; Sun et al. 2018). We suggest two complementary explanations. First, the PCo1 axis captures structural variation in community composition but does not necessarily track the physiological activity of functionally relevant taxa. In other words, community presence does not equal community activity, as demonstrated for methanogen and methanotroph communities in peatlands (Martí et al. 2015). Further, to minimise disturbance to permanent plots, peat sampling was restricted to *c.* 5 cm below the *Sphagnum* surface; a pragmatic choice given the long-term nature of the experiment (i.e. to avoid structural damage to the plots). Yet, it warrants consideration when interpreting the SEM results. Methanogenic archaea, which are most abundant around 35 cm depth in ombrotrophic peats (Asemaninejad et al. 2019), were correspondingly rare in our dataset (*c.* 0.01% of prokaryotic reads), limiting our ability to characterise the archaeal community. Similarly, ericoid mycorrhizal and lignocellulose-degrading fungi exhibit depth optima spanning 0–40 cm (Lamit et al. 2021), with peak fungal richness concentrated near the water table (Asemaninejad et al. 2017). These zones are not fully represented by our sampling design. The absence of detectable microbial mediation of CH₄ fluxes should therefore be interpreted in light of this depth constraint, rather than as evidence that the deeper microbial community is uninvolved in CH_4_ cycling at this site.

The divergence between the bacterial and fungal SEMs may reflect differences in how bacterial and fungal communities respond to vegetation change. In the bacterial model, PFT treatment significantly affected community composition, consistent with the idea that changes in plant functional composition can influence methane-cycling microbial communities (Robroek et al. 2015; Shao et al. 2022). Although our SEMs do not provide definitive evidence for mediation, this pattern suggests that bacterial community responses may contribute to the relationship between PFT treatment and CH_4_ fluxes. In contrast, fungal community composition showed little response to PFT treatment, causing the direct influence of microhabitat on CH₄ fluxes to be more apparent in the fungal model. Together, these results suggest that vegetation effects on methane dynamics are more closely associated with bacterial than fungal community variation, whereas hydrological differences between microhabitats remain an important direct driver of CH_4_ emissions.

Our results highlight a key role of vascular plant photosynthesis in summer carbon uptake in ombrotrophic peatlands. Under projected climate warming, vascular plant cover is expected to expand at the expense of *Sphagnum* mosses (Dieleman et al. 2015; Lyons et al. 2020), which sustain carbon uptake over a longer seasonal window (Glenn et al. 2006) and chemically suppress litter decomposition across PFTs (Mao et al. 2018). While expanding vascular plant cover may initially enhance summer CO_2_ uptake, the concurrent stimulation of peat decomposition via priming effects, increased aerenchymatous CH₄ transport, and reduced *Sphagnum* inhibition of decomposition is likely to erode the annual carbon sink function over time (Ward et al. 2013; Walker et al. 2016). The net outcome will depend critically on the interactions among warming, nitrogen deposition, and drought, which modulate the direction and magnitude of PFT effects on peatland carbon fluxes (Gong et al. 2024; Sterk et al. 2023). Fully resolving these dynamics requires year-round measurements that capture both plant-driven and microbially mediated carbon cycling throughout the seasonal cycle, and deep-peat sampling to characterise the functional microbial guilds most relevant to long-term carbon storage.

To conclude, we show that summer CO_2_ uptake in this ombrotrophic bog is primarily driven by vascular plant photosynthesis, with progressive loss of NEE following PFT removal. CH_4_ emissions were governed by microhabitat rather than by plant community composition, consistent with a dominant hydrological control on methanogenesis at the prevailing water table depth. Microbial community structure responded to microhabitat and, for bacteria, to the most intensive vegetation manipulation, but did not mediate carbon fluxes in the SEM framework. The latter we attribute primarily to the shallow sampling depth constraining detection of the key functional guilds. Our findings emphasise the importance of vertical stratification and seasonality in studies of peatland carbon cycling, and underscore the need for integrated, year-round approaches that couple plant and microbial community assessments across the full peat profile.

## Data Availability

Bagdonaitė, A., Thomas, C.L., Telgenkamp, Y., Robroek, B.J.M., 2026, Plant functional type composition drives summer carbon cycling in an ombrotrophic peatland, https://doi.org/10.17026/LS/6QU1CF, DANS Data Station Life Sciences.
